# Oxidative Depolymerization of Alkaline Lignin from Pinus Pinaster by Oxygen and Air for Value-Added Bio-Sourced Synthons

**DOI:** 10.3390/polym13213725

**Published:** 2021-10-28

**Authors:** Martin Camus, Olivia Condassamy, Frédérique Ham-Pichavant, Christelle Michaud, Sergio Mastroianni, Gérard Mignani, Etienne Grau, Henri Cramail, Stéphane Grelier

**Affiliations:** 1 CNRS, University Bordeaux, Bordeaux INP, LCPO, UMR 5629, 33600 Pessac, France; martin.camus92@gmail.com (M.C.); Olivia.condassamy@mauvilac.com (O.C.); frederique.pichavant@enscbp.fr (F.H.-P.); etienne.grau@u-bordeaux.fr (E.G.); 2 Rayonier AM France Innovation, 33174 Gradignan, France; Christelle.Michaud@rayonieram.com; 3 Research and Innovation Center of Lyon, Solvay, 85 Avenue des Frères Perret, 69192 Saint Fons, France; sergio.mastroianni@solvay.com; 4 International Open Innovation, 56580 Kerfily en Crédin, France; gerard.mignani8@gmail.com

**Keywords:** lignin, oxidative depolymerization, oxygen, air, oligomers, hydrosoluble lignin

## Abstract

In this work, an efficient 3-step process targeting the chemical modification and purification of lignin oligomers from industrial alkaline lignin is described. The oxidative depolymerization process of alkaline lignin with O_2_ or Air pressure, without use of metal catalyst, led to the production of two fractions of lignin oligomers named ‘precipitated lignin’ and ‘hydrosoluble lignin’ with 40% and 60% yield, respectively. These fractions were characterized with a wide range of methods including NMR spectroscopy (31P, 2D-HSQC), SEC (in basic media), FTIR. NMR analyses revealed the presence of carboxylic acid functions at a ratio of 1.80 mmol/g and 2.80 mmol/g for the precipitated and hydrosoluble lignin, respectively, values much higher than what is generally found in native lignin (between 0.2 and 0.5 mmol/g). SEC analyses revealed the formation of low molar masses for the precipitated (2200 g/mol) and hydrosoluble fractions (1500 g/mol) in contrast to the alkaline lignin (3900 g/mol). It is worth noting that the hydrosoluble fraction of lignin is soluble in water at any pH. Both processes (oxygen and air) were successfully scaled up and showed similar results in terms of yield and functionalization.

## 1. Introduction

Biomass represents a primary renewable feedstock for the manufacture of polymeric materials. After cellulose, lignin is the most abundant biopolymer on earth. On an annual basis, the production of lignin is estimated in the range of 50 million tons [1]. In this concern, technical lignins represent a very promising resource for the production of aromatic chemicals and phenolic-containing [2,3] polymers.

Lignin is a complex polyphenolic biopolymer whose structure and properties basically depend on the raw material, growing conditions of the plant and extraction methods used [4]. Despite this challenge to isolate lignin, pulping industries are developing processes to deconstruct biomass which are classified according to the pH and solvents used as typically acidic, alkaline or organosolv processes [5].

The valorization of technical lignin for valuable organic feedstocks is extensively studied and represents a real challenge to substitute fossil resources. Oxidative processes have been intensively studied leading to a wide variety of structures with different molar masses ranging from monophenol to oligomers and bearing chemical functions such as alcohol or carboxylic acid moieties [6]. The reactivity of several oxidants, namely chlorine dioxide, ozone, dimethyldioxirane and hydrogen peroxide were investigated by Sun and Argyropoulos [7]. The authors have shown that guaiacyl units were the most reactive and led mainly to carboxylic acid functions. Among the different proposed pathways, catalytic degradation into aromatics such as vanillin [8,9,10] and thermochemical treatment for carbon material treatment lead to high value chemicals or materials [11,12]. In numerous applications such as concrete plasticizer [13] or lignin-based foams [14], partially depolymerized and functionalized lignin could bring new opportunities for automotive manufacturing or building construction.

In this present study, an efficient oxidative depolymerization process of technical lignin produced by alkaline purification step in local biorefinery was implemented to produce functional and low molar mass oligomers of lignin. The experimental oxidative conditions of lignin treatment have been optimized and the structure of the oligomers so-formed elucidated.

## 2. Materials and Methods

### 2.1. Materials

Two industrial alkaline lignins from Pinus pinaster supplied by Rayonier Advanced Materials (RYAM, Tartas, France) were produced from same extracted alkaline liquor. After ammonium bisulfite cooking process, an alkaline extraction was applied at different temperatures to remove residual lignin from the crude pulp. Lignin with low sulfur content was extracted from this alkaline liquor by two different acidic processes. The first one at lab scale was performed by adding a hydrochloric solution until pH 1 (LN1) and the second one at pilot scale by using sulfuric acid (LN2). The chemical structure and thermal properties of these alkaline lignins were compared with those of a kraft lignin from Pinus pinaster (supplied by Smurfit Kappa Cellulose du Pin, Biganos, France). Such a kraft lignin was also extracted by precipitation in acidic medium.

### 2.2. Lignins Oxidative Depolymerization Procedure

Alkaline lignins (2.35 g) were solubilized in basic water solution at the concentration of 40 g/L and NaOH/Lignin weight ratio was kept at 0.6 to avoid the precipitation of oxidized lignin during the process, as the carboxylic acid functions consume alkali. The mixture was then poured and stirred in a stainless steel reactor without addition of any metal catalyst. Oxidant medium (O_2_ or air), temperature and contact time were varied to determine the effect of these parameters on lignin characteristics. At the end of the reaction, the pressure drop was monitored and the reaction was cooled down to room temperature as well as the final pH of the solution. Due to the formation of carboxylic acid units, the pH decreased from 13 to 7.5.

### 2.3. Purification Procedure

The product was isolated from the oxidative media by precipitation in acidic media. A hydrochloric solution (1 M) was slowly added to this solution under stirring until the pH value decreased to 1 and 2. The supernatant was removed by centrifugation (5500 g, 15 min, 10 °C) and the precipitated fraction (LNP) recovered, washed twice with acidified water (pH = 2) and then freeze-dried to obtain a yield of 58%. The supernatant fraction was then extracted with dichloromethane (DCM) to remove low molar mass phenolic structures. The DCM solution was then isolated with a yield around 2% and evaporated to give the organosoluble fraction (OF). The aqueous phase was evaporated under vacuum, placed in methanol to eliminate residual salts coming from alkaline liquor or the precipitation process. Salts were removed by centrifugation (5500× *g*, 15 min, 10 °C) and the methanol was then slowly evaporated at low temperature under vacuum. This hydrosoluble lignin (LNW) was soluble in water, whatever the pH. The yield of this fraction was around 40 wt.%. A volatile fraction was also recovered but not quantified and is mainly composed of carbon dioxide due to partial decarboxylation of carboxylic acid groups produced by oxidation of the lignin.

### 2.4. Lignin Characterizations

The ratio of lignin contained in alkaline liquor was determined by Klason and Acid soluble Lignin (ASL) [15] standard methods widely described in the literature. Mineral contents of lignin samples were determined by gravimetric analyses after burning the lignins at 575 ± 5 °C [16]. Other components (sugars and additives) were determined by difference.

The purity of lignin from alkaline liquor was obtained by UV spectroscopy method with a Lambda 18 spectrophotometer from PerkinElmer (Waltham, MA, USA). Solutions of alkaline liquor were prepared by suitable dilution (typically 1.10–3% *w*/*w*) with distillated water and absorbance at 280 nm was recorded at three different times. Average of values were calculated and concentration of lignin was determined by Beer-Lambert law with molecular extinction coefficient ε = 25 L/g/cm [17].

The metallic ratio has been analyzed with an ICP OES AGILENT 5100 (Santa Clara, CA, USA). First, the lignin sample (around 1 g) was mineralized with nitric sulfur and was then taken back into a 20 mL vial with sulfuric acid 5%.

### 2.5. Instruments Methods

Fourier transform Infrared Spectroscopy (FTIR). Analyses were performed on a thermo Nicolet spectrometer Avatar 370 instrument (Waltham, MA, USA) by direct transmittance [18]. Measurements were run for 32 scans in the spectral range between 4000 and 400 cm^−1^ with 4 cm^−1^ resolution (Figure S1).

Nuclear Magnetic Resonance (NMR) Spectra experiments were performed at 298 K on a Bruker Avance 400 spectrometer (Billerica, USA) operating at 400 MHz. Hydroxyl groups of lignins were quantified by quantitative 31P NMR (Figure S2). Spectra were obtained using procedures previously described by Argyropoulos [19]. Briefly, 2-chloro-4,4,5,5-tetramethyl-1,3,2-dioxaphospholane (TMDP) as phosphorylating reagent and endo-*N*-hydroxy-5-norbornene-2,3-dicarboximide as internal standard [20]. Accurate amounts of 40–45 mg of a dried lignin sample, 2–10 mg of internal standard and 1 mg of relaxation agent (chromium(III) acetylacetonate) were dissolved in 500 μL of anhydrous pyridine/CDCl3 mixture (1.6/1 *v*/*v*). 500 μL of dried DMF were added when the mixture is not soluble. Then, 100 μL of phosphorylating reagent (TMDP) were added and the reaction was carried out for 1 hour. Finally, 400 μL of the mixture were transferred into a 5-mm NMR tube for subsequent NMR acquisition. The content of hydroxyl groups in lignin fractions was obtained by integration of the following spectral regions: aliphatic hydroxyls (150.0–145.5 ppm), condensed phenolic units (144.7–140 ppm), guaiacyl (G) phenolic hydroxyls (140.2–138.6 ppm), p-hydroxyphenyl (H) phenolic hydroxyls (138.4–136.4 ppm) and carboxylic acids (136–134 ppm).

2D HSQC NMR spectra were recorded at 298 K on a Bruker Avance 400 spectrometer (Billerica, MA, USA) operating at 400 MHz (Figure S3). Lignins (70 mg) were dissolved in 0.65 mL of dimethyl sulfoxide (DMSO)-d6. The central solvent peak was used as internal reference (DMSO δC/δH 39.5/2.5). The HSQC (heteronuclear single quantum coherence) experiment used was Bruker’s “hsqcetgpsisp2.2” pulse program. Signal assignment was made by correlation with acetylated or milled wood lignins found in literature [21,22,23].

Size Exclusion Chromatography (SEC). The molar mass distribution of lignins was determined by alkaline SEC with TSK gel G3000 + G4000 + G3000 PW columns from Tosoh Bioscience (kinf of Prussia, PA, USA), in alkaline solution (pH = 12) as eluent with a flow rate of 1 mL/min, UV detection at 280 nm and calibration with sodium-polystyrene sulfonate standards, according to the procedure described by Gosselink [24]. M_n_, M_w_ and dispersity Ð (M_w_/M_n_) values were calculated by mean of cirrus program.

## 3. Results and Discussion

### 3.1. Characteristics of Technical Lignins

In this study, two different lignin samples have been provided by RYAM. The latter were produced by acidic precipitation from the same extracting liquor, either with hydrochloric acid (LN1) or with sulfuric acid (LN2). The composition of these samples is given in Table 1. The lignin purity of these different samples showed close results with a purity (Klason + ASL) around 95% for LN1 and 97% for LN2. The remaining 5% or 3% can be attributed to sugar derivatives strongly bonded to lignin and inorganic compounds (around 0.5% of ashes after thermal degradation of lignin samples). These side species were similar in quantity and composition for kraft lignin (Table 1). The inorganic compounds were extracted by acidic precipitation, firstly with carbon dioxide and then with a mineral acid to the complete precipitation of lignin. By following this procedure, the kraft lignin purity is close to 98%. For the following experiments, all mass calculations will take into account the dryness of the sample and the lignin purity.

The processes applied to purify the lignin samples modified their chemical composition and function ratios. Data are presented in Table 1. The precipitation of kraft lignin is sensitive to pH, probably due to the presence of carboxylic groups on the lignin [25]. 

The phenolic group contents of LN1 and LN2 are lower to the one of kraft lignin. These results are consistent with the higher molar mass of lignin due the alkaline extraction performed by Rayonier at lower temperature in comparison to the kraft cooking.

### 3.2. Influence of O_2_ Pressure on the Oxidative Depolymerization of Alkaline Lignin

The effect of O_2_ pressure on the oxidative depolymerization of lignin was first investigated with a temperature of 180 °C. According to literature [26,27], it is anticipated that oxygen pressure promotes strong chemical modifications on lignin skeleton with the formation of carboxylic acid functions. Indeed, the impact of oxygen pressure (2, 5 and 10 bar) on the oxidative depolymerization of lignin was first investigated and data are shown in Table 2. A decrease of the lignin molar mass is clearly observed with the increase of O_2_ pressure. In all cases, precipitated and hydrosoluble fractions were recovered. The mass balance allowed us to determine the volatile fraction (y_Pr_ + y_HS_ + y_vol_ = 1). Experiments also showed that a higher temperature produces a decarboxylation of lignin structure as demonstrated by the formation of carbon dioxide (determined by limewater test) and reduced yields of precipitated and hydrosoluble fractions. Both fractions were then analyzed by SEC and 31P NMR. As can be noticed in Table 2, the pressure of O_2_ has a strong impact on the concentration of the various functional groups of lignin. The higher the O_2_ pressure, the higher the concentration of COOH functions both onto the precipitated and hydrosoluble lignin fractions. During the oxidative treatment, the concentration of aliphatic alcohol functions decreases from 1.32 mmol/g to 0.36 mmol/g for the precipitated fraction and to 0.16 mmol/g for the hydrosoluble one. The same trend is observed for the condensed and guaiacol moieties. In contrast, the oxidative treatment brings new carboxylic groups onto the lignin backbone as the carboxylic acid ratio increases from 0.27 mmol/g to 2.27 mmol/g for the precipitated lignin and to 1.64 mmol/g for the hydrosoluble one. FTIR analyses of LN1 and oxidative fractions, LNP and LNW (Figure S1) confirm the increase of carboxylic acid (C=O band at 1710 cm^−1^ and the decrease of hydroxyl groups (broad O–H band around 3300 cm^−1^). The SEC traces (Figure S4) show a little decrease of the lignin molar mass after the oxidative treatment evidenced by a reduction of lignin molar mass. As expected, the higher the O_2_ pressure, the lower the average molar mass values of the precipitated and hydrosoluble fractions (Table 2).

### 3.3. Influence of Temperature on the Oxidative Depolymerization of Alkaline Lignin

The amount of volatile fraction observed during the oxidative treatment suggested that an increase of the O_2_ pressure was responsible of lignin decarboxylation and of a low depolymerization yield. The effect of the temperature was also evaluated on the oxidative depolymerization of lignin together with the production of carboxylic functions. Two temperatures were compared, 180 °C (previously discussed) and 120 °C for a same oxygen pressure (10 bar of O_2_); the results are reported in Table 3. At 120 °C, the decarboxylation phenomenon is less and the volatile fraction remains limited to 1%. Consequently, the yields obtained in precipitated and hydrosoluble fractions are much higher at 120 °C. In addition, a lower amount of carboxylic groups is logically formed at 120 °C in comparison to 180 °C. Indeed, the ratio of COOH groups of the precipitated and hydrosoluble lignin fractions obtained at 120 °C are equal to 1.80 mmol/g and to 1.15 mmol/g, respectively. It is worth noting that, at 120 °C, aliphatic alcohol and guaiacyl units are less impacted by the oxidative treatment. Finally, the temperature was found to be an important parameter to tune the yields of precipitated and hydrosoluble lignin fractions as well as the ratio of acidic functions brought by these fractions.

These first investigations clearly show that a compromise must be found in order to functionalize the lignin fractions without generating an important decarboxylation phenomenon. The conditions 120 °C/10 bar of oxygen seem to be the most suitable for carrying out an oxidative depolymerization, with an overall yield close to 100%.

Another alkaline lignin produced by using sulfuric acid (LN2) was also treated under the same conditions; results are presented in Table 4. Alike previous data, the yields of lignin fractions were close to those obtained previously: 40% for the precipitated fraction and 58% for the hydrosoluble one, at 120 °C and 10 bar of oxygen. These conditions were also found the best ones to obtain the highest ratio of carboxylic functions. The ratio of carboxylic acid functions was higher in the case of LN2 versus LN1 (1.87 mmol/g and 2.76 mmol/g for the precipitated and hydrosoluble fraction, respectively), suggesting that processes implemented for the production of lignin have an impact on the oxidative depolymerization. The phenolic and aliphatic alcohol ratios, obtained after the oxidative treatment, were relatively close between the two alkaline lignin samples. 

The SEC traces (Figure S5), showed similar features as for LN1 and LN2; the oxidative depolymerization process yielding an important decrease of molar masses. However, this decrease was found more important with LN2, allowing us to obtain oligomers with higher carboxylic acid ratios.

**Figure 1:**
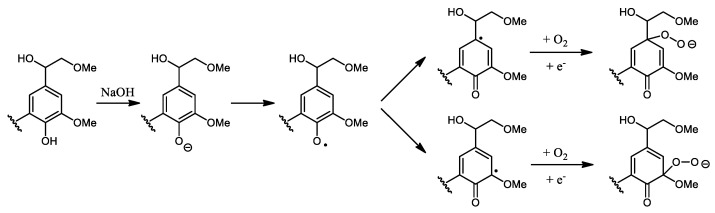
Insertion of oxygen on several positions of the phenolic structure of lignin.

The oxidative depolymerization of lignin is a very complex process that involves different possible mechanisms. Many research groups investigated the impact of oxygen and hydrogen peroxide as oxidative agents onto the lignin structure [26,28,29,30,31]. In alkaline medium, the phenol units are first deprotonated then, the phenoxy radical, formed by an electron transfer on oxygen, is stabilized by resonance. An electron acceptor as oxygen or oxygen peroxide can be fixed on several positions of the phenolic structure as described in Figure 1.

**Figure 2:**
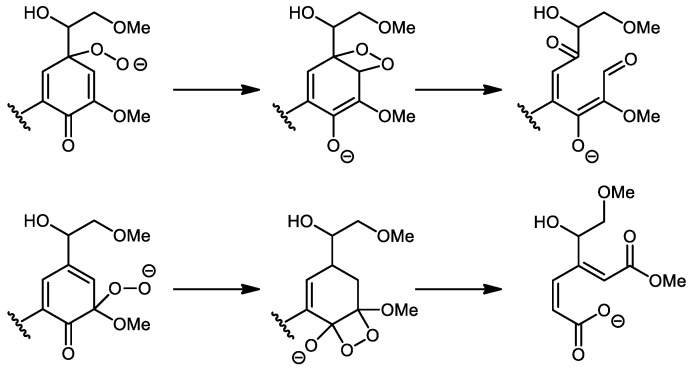
Oxidative ring-opening of the aromatic structures.

The second step suggested the formation of carbonyl and carboxylic acid moieties, depending on the position of the peroxide function on the aromatic skeleton. A ring-opening of the aromatic structures may occur as indicated in Figure 2.

The 2D-HSQC NMR (supporting information, Figure S3) also demonstrated the depolymerization of lignin with the appearance of new aromatic structures and the disappearance of functional units like guaiacyl and phenol. The oxidative depolymerization enabled the formation of new functions in accordance with the proposed mechanisms and the 31P NMR analyses. It provides an insight to explain why one fraction (precipitated fraction) remains insoluble in water while the other (hydrosoluble fraction) is not. Indeed, the oxidative reagent seems to impact dramatically the aromatic structure of lignin by creating carboxylic acid functions thus improving the water-soluble ability of lignin oligomers.

### 3.4. Oxidative Depolymerization of Alkaline Lignin with Air

The results discussed above demonstrated that oxygen is a very efficient oxidant for the lignin depolymerization and functionalization. However, with the objective to reduce the economic and environmental impacts of this process, the use of Air instead of pure O_2_ was investigated.

A preliminary experiment was performed to determine the impact of air pressure with respect to the carboxylic acid functionalization ratio. 

When 10 bar of air at 120 °C was used, the hydrosoluble fraction represented around 20% of the total yield, value to be compared to 60% with the same pressure of oxygen.

Following this preliminary test, several experiments were carried out with an air pressure of 10 bar, 20 bar, 30 bar, 40 bar, 50 bar to evaluate the pressure required to obtain the best yield in functionalized fractions of lignin, while limiting the formation of volatile fraction. 

**Figure 3:**
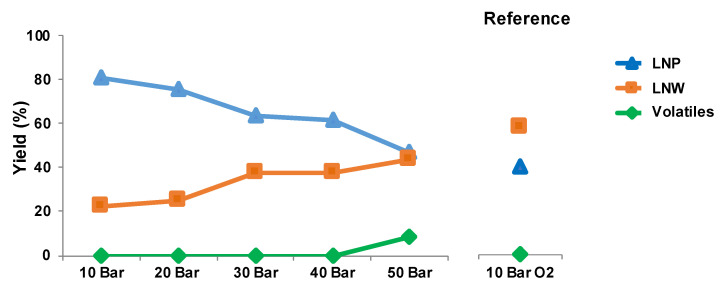
Yields obtained during oxidative depolymerization of LN2 with different air pressure compared to 10 bar oxygen pressure.

As expected, an increase in air pressure improved the yields of the LNW fraction at the cost of the LNP one (Figure 3). At 50 bar of air, the yields in both fractions are close to the ones obtained with 10 bar of pure oxygen: 45% for the precipitated and 45% for hydrosoluble fraction compared to 40% and 60%, respectively with oxygen alone. However, a higher air pressure confirms the formation of volatiles by decarboxylation phenomenon, reducing the overall yield of the oxidative depolymerization reaction.

**Figure 4:**
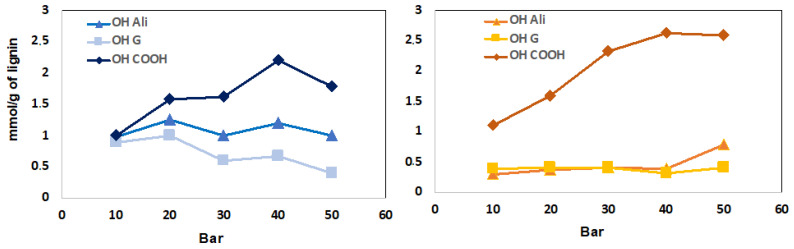
Evolution of carboxylic acid (COOH), guaiacol (G) and aliphatic alcohol (Ali) units according to the air pressure used for LNP (**left**) and LNW (**right**).

Regarding the carboxylic acid functionalization ratio, an increase of the air pressure leads to a gradual decrease of the content of aliphatic alcohol and phenolic functions of the precipitated and hydrosoluble lignin fractions (Figure 4). In comparison to experiments performed with pure O_2_, similar functionalization ratios were obtained 1.87 mmol/g and 2.60 mmol/g of COOH functions for the precipitated and hydrosoluble lignin fractions, respectively. As can be seen in Figure 4, an optimum of carboxylic acid amount was obtained at 40 bar. Beyond this pressure, a decarboxylation phenomenon occurs generating the formation of carbon dioxide.

**Figure 5:**
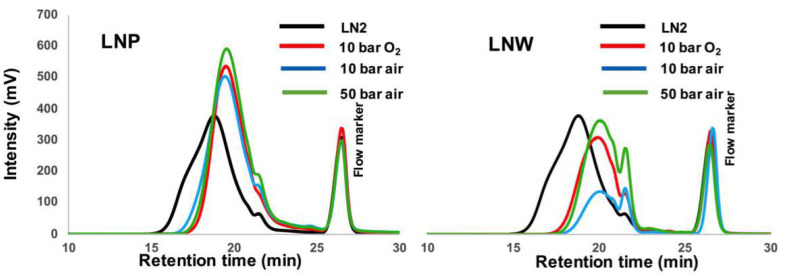
SEC chromatograms of LNP and LNW after oxidative depolymerization of LN2 (black) with different air or oxygen pressure, at 120 °C.

The SEC analyses (Figure 5) showed similar chromatograms as the ones obtained previously for both fractions. First, the molar masses similarly decrease, proving that the oxidizing species (oxygen or air) had the same impact while the quantities used are different. However, chromatograms showed little differences, with the appearance of species of lower molar masses when the oxidative depolymerization was performed under air. These experiments showed that a higher overall pressure created smaller oligomers with a higher decarboxylation of lignin oligomers. 

## 4. Conclusions

An efficient oxidative process, involving O_2_ or Air pressure without any catalyst, to depolymerize and functionalize lignin was developed. Several experimental conditions (O_2_ or Air pressure and temperature notably) have been tested in order to obtain the best yields of lignin fractions and functionalization ratios. This oxidative depolymerization produced two types of lignin fractions, one soluble in water whatever the pH (hydrosoluble fraction) and a second that precipitates (precipitated fraction). The optimized conditions to obtain the better conversion of this lignin were 10 bar of oxygen with a reaction temperature of 120 °C. In these conditions, both fractions revealed lower molar masses in comparison to native lignin together with a high ratio of carboxylic acid functions and sufficient phenolic moieties to maintain antioxidant properties. For example, the hydrosoluble fraction was utilized as polyanion in layer by layer assemblies to provide UV-protection [32]. Further developments describing the use of these carboxylic acid-rich lignin fractions will be described in forthcoming papers.

## Figures and Tables

**Table 1:** Main characteristics of Rayonier AM alkaline lignins.

	LN1	LN2	Kraft Lignin *
Purity (%) (Klason + ASL)	95	97	98
Ashes (%)	1	0.5	0.5
Mn (g/mol)	3400	3900	2000–20,000
Ð	4.5	3.6	2–4
Aliphatic OH ratio (mmol/g)	1.32	1.41	1.65
Phenolic ratio (mmol/g)	2.08	1.61	3.25
COOH ratio (mmol/g)	0.27	0.36	0.17

* Kraft lignin provide by local mill.

**Table 2:** Effect of oxygen pressure on the oxidative depolymerization of LN1 (180 °C, 1 h); precipitated lignin (LNP), hydrosoluble lignin (LNW).

	Lignin Fraction			
O_2_ Pressure (Bar)		2	5	10
Oxygen Equivalent		5	12	24
Volatile ratio (%)		0	9.7	32.3
Yield (%)	LNP	69	41.3	12.2
COOH content (mmol/g)		1.65	2.04	2.27
Mn (g/mol)		1700	1300	900
Ð		1.9	2.5	2.4
Yield (%)	LNW	31.2	49	55.5
COOH content (mmol/g)		0.93	1.40	1.64
Mn		1700	3100	2000
Ð		1.8	1.2	1.4

**Table 3:** Effect of temperature on the oxidative depolymerization of LN1 (10 Bar O_2_, 1 h); precipitated lignin (LNP), hydrosoluble lignin (LNW).

	Lignin Fraction		
Temperature		180	120
Volatile ratio (%)		32.3	1
Yield (%)	LNP	12.2	37.5
COOH content (mmol/g)		2.27	1.80
Mn (g/mol)		900	2800
Ð		2.4	2.1
Yield (%)	LNW	55.5	59.5
COOH content (mmol/g)		1.64	1.15
Mn		2000	2200
Ð		1.4	2.0

**Table 4:** Oxidative depolymerization of different lignin sample (10 Bar O_2_, 1 h); precipitated lignin (LNP), hydrosoluble lignin (LNW).

	Lignin Fraction		
Lignin sample		LN1	LN2
Volatile ratio (%)		1	0
Yield (%)	LNP	37.5	40
COOH content (mmol/g)		1.80	1.87
Mn (g/mol)		2800	2200
Ð		2.1	1.7
Yield (%)	LNW	59.5	58
COOH content (mmol/g)		1.15	2.76
Mn (g/mol)		2200	1500
Ð		2.0	1.5

## Data Availability

Not applicable.

## Supplementary Materials

The following are available online at https://www.mdpi.com/article/10.3390/polym13213725/s1, Figure S1: FTIR analysis of LN1 (in black), LNP (blue) and LNW (orange); Figure S2: 31P NMR of LN1; Figure S3: 2D HSQC NMR of LN1 before and after oxidative depolymerization with a focus on aliphatic (on the right) and aromatic (on the left) areas; Figure S4: SEC chromatogram of LN1 lignin (blue curve) and the influence of the oxygen pressure at 180 °C on LNP and LNW molar masses.; Figure S5: SEC chromatogram of LN1 (red curve) and LN2 (black curve) before oxidative depolymerization and the LNP and LNW fractions obtained from these both lignin respectively (all experiments was performed with 10 bar O_2_ and 120 °C).
