# Supplementary material for: Oxidative Depolymerization of Alkaline Lignin from Pinus Pinaster by Oxygen and Air for Value-Added Bio-Sourced Synthons
Source: Polymers (Basel). 2021 Oct 28;13(21):3725. doi: 10.3390/polym13213725 (PMC8587034; doi:10.3390/polym13213725)
Supplement: Supplementary file 1 [file polymers-13-03725-s001.zip › polymers-1393782-supplementary.pdf]

Supplementary Material

Oxidative depolymerization of alkaline lignin from Pinus pinaster by oxygen and air for value-added bio-sourced synthons

Martin Camus[a], Olivia Condassamy[a], Frédérique Ham-Pichavant[a], Christelle Michaud[b], Gérard Mignani[c], Sergio Mastroianni[c], Etienne Grau[a], Henri Cramail[a*], Stéphane Grelier[a*]

[a] Univ. Bordeaux, CNRS, Bordeaux INP, LCPO, UMR 5629, F-33600, Pessac, France

[b] Rayonier AM France Innovation 33174 Gradignan, France

[c] Solvay, Research and Innovation Center of Lyon, 85 avenue des Frères Perret 69192 Saint Fons, France

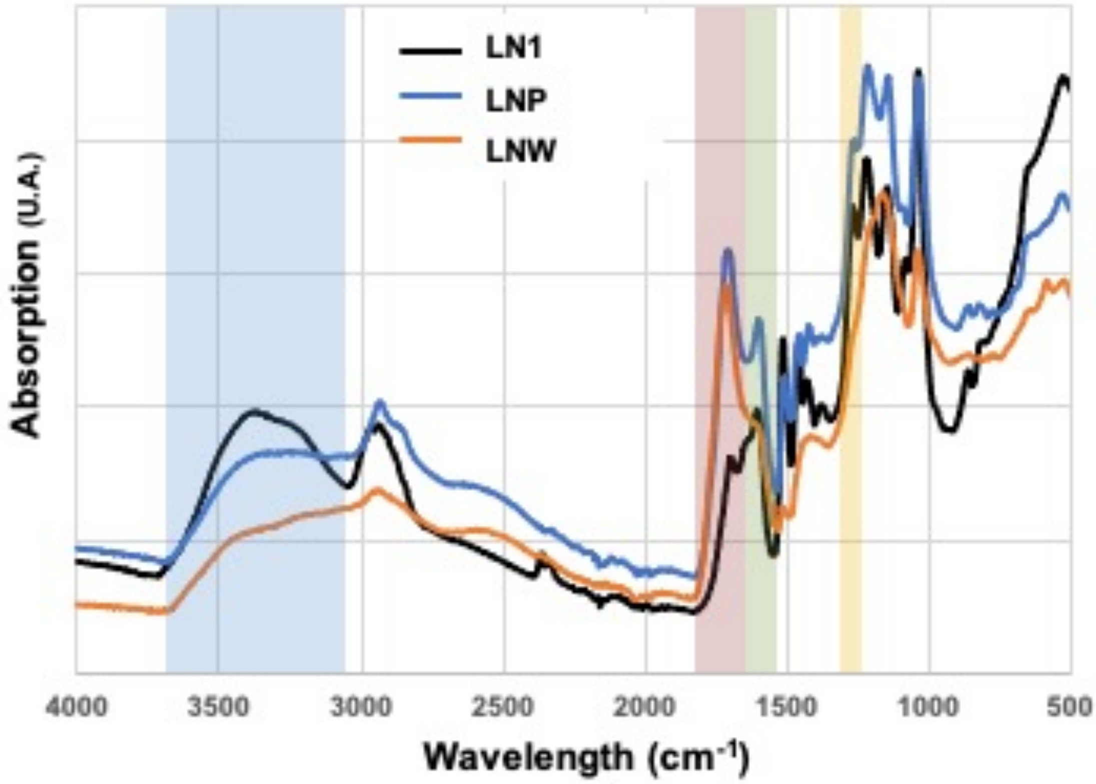

Figure S1: FTIR analysis of LN1 (in black), LNP (blue) and LNW (orange)

O-H stretching of alcohols

C=O stretching of carbonyls

C=C stretching of aromatics

C-C, C-O stretching of gaïacyl

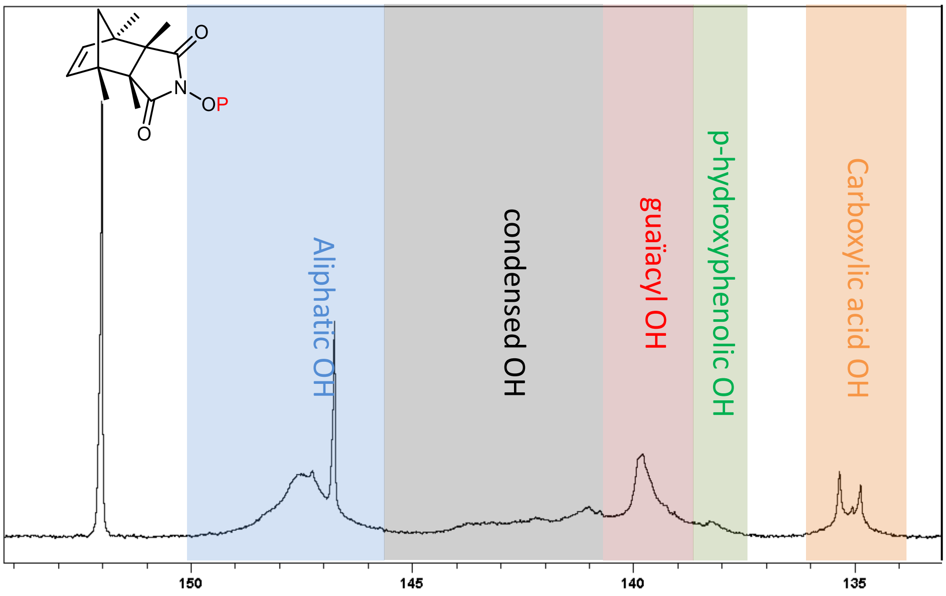

Figure S2. 31P NMR of LN1.

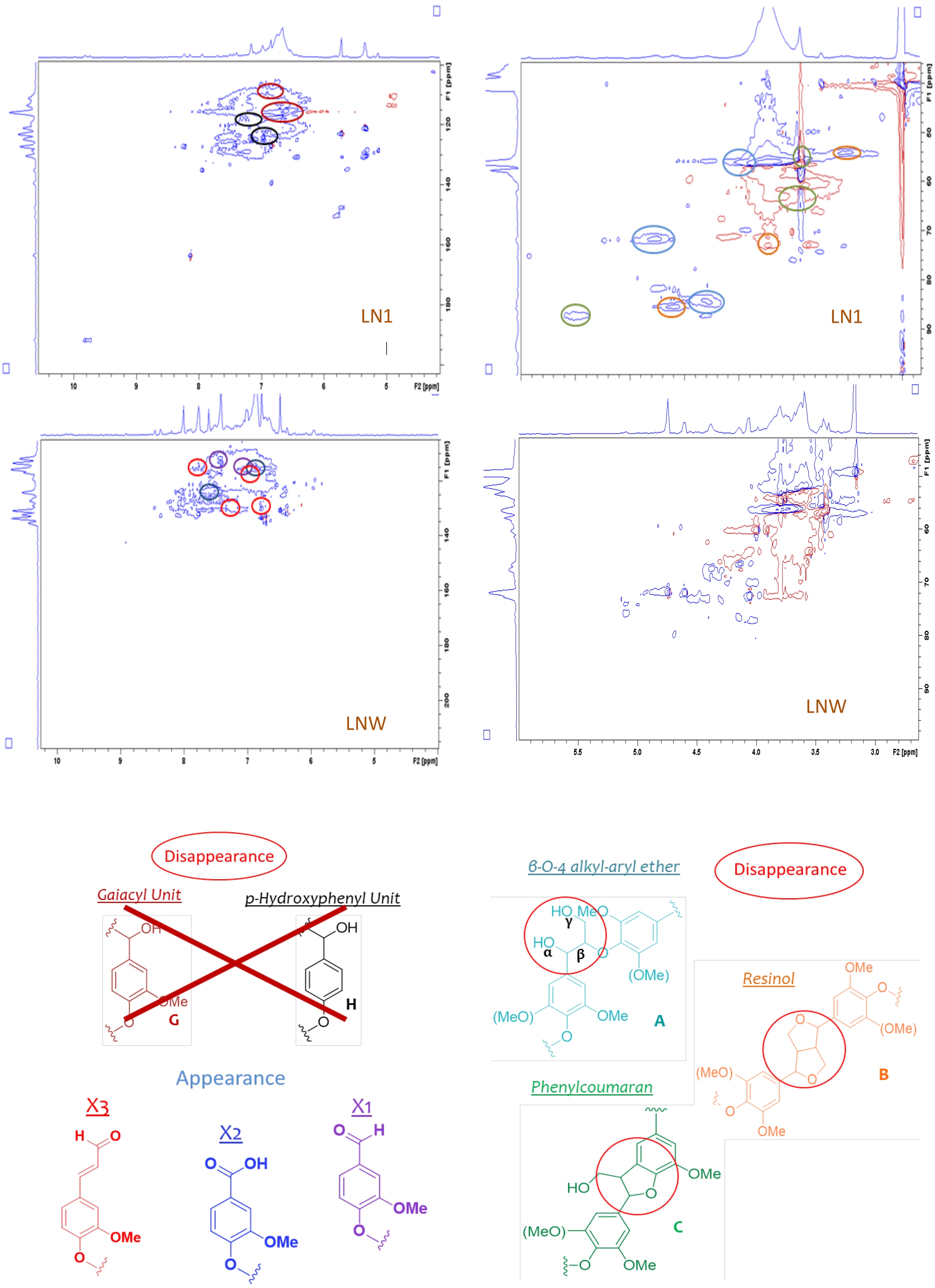

Figure S3: 2D HSQC NMR of LN1 before and after oxidative depolymerization with a focus on aliphatic (on the right) and aromatic (on the left) areas

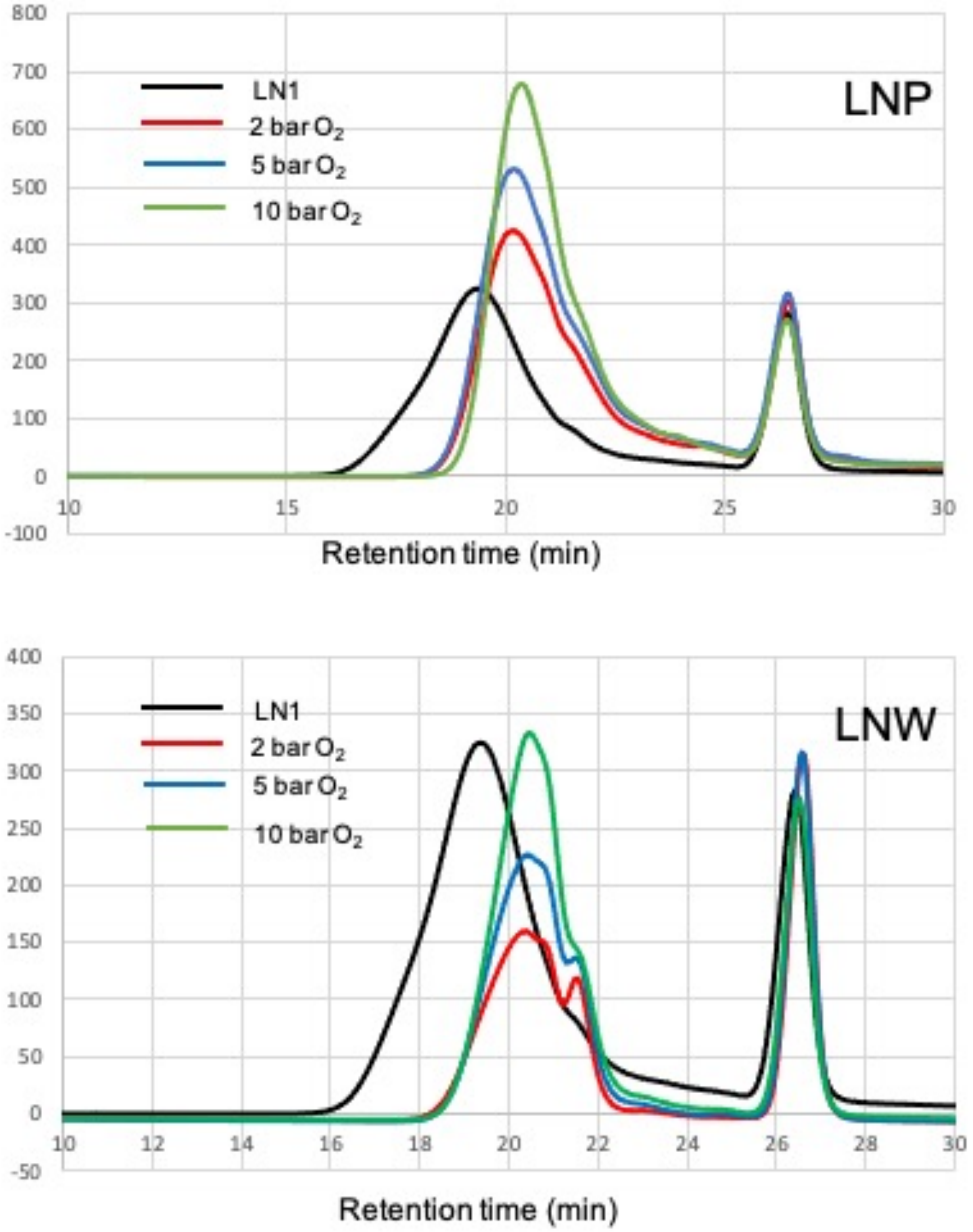

Figure S4 SEC chromatogram of LN1 lignin (blue curve) and the influence of the oxygen pressure at 180°C on LNP and LNW molar masses.

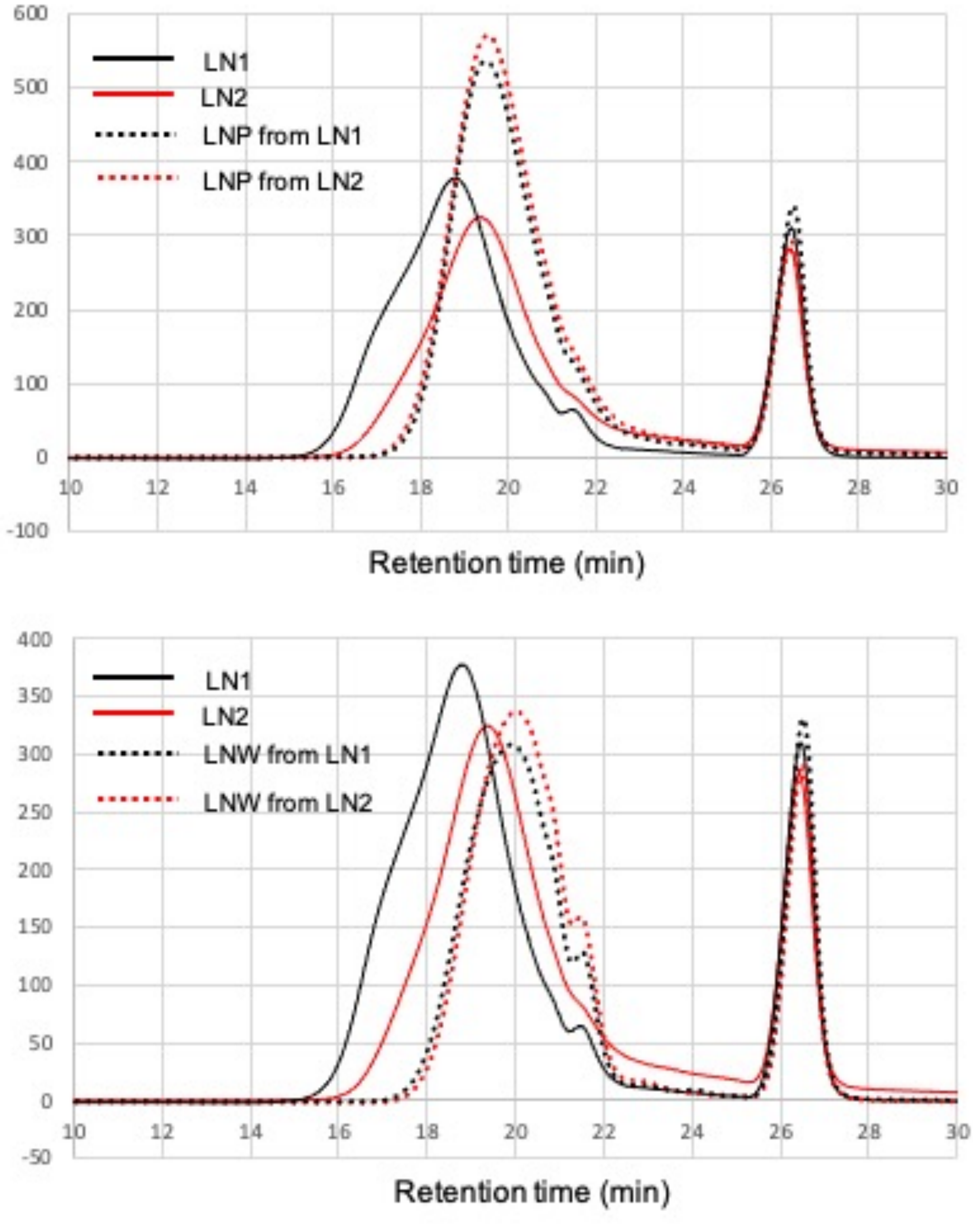

Figure S5: SEC chromatogram of LN1 (red curve) and LN2 (black curve) before oxidative depolymerization and the LNP and LNW fractions obtained from these both lignin respectively (all experiments was performed with 10 bar O_2 and 120°C)
